# Cross-country assessment of the unique contributions of psychological factors to vaccination: Perspectives on the COVID-19 pandemic

**DOI:** 10.1177/13591053241266592

**Published:** 2024-07-31

**Authors:** Peter Adu, Tosin Popoola, Naved Iqbal, Anja Roemer, Sunny Collings, Clive Aspin, Oleg N Medvedev, Colin R Simpson

**Affiliations:** 1 Victoria University of Wellington, New Zealand; 2 The University of Newcastle, Australia; 3 Jamia Millia Islamia, India; 4 Massey University, New Zealand; 5 University of Waikato, New Zealand; 6 The University of Edinburgh, UK

**Keywords:** compassion, COVID-19, optimism, psychological, religiosity, vaccination

## Abstract

We have identified the most relevant and significant psychological factors in relation to COVID-19 vaccination attitudes in Ghana, Germany, New Zealand and India. This study recruited 1822 participants from the general populations of India (*n* = 411), New Zealand (*n* = 413), Ghana (*n* = 523) and Germany (*n* = 475) to participate in a cross-sectional online survey. After controlling for the country of residence, individual psychological factors played a significant role in shaping attitudes towards COVID-19 vaccination. The results also revealed strong direct predictors that explained significant portions of the variance in the COVID-19 vaccination attitudes. Positive affect emerged as the strongest contributor in Ghana (7%), while self-compassion strongly influenced COVID-19 vaccination attitudes in India (66%). Dispositional optimism was the strongest predictor in New Zealand (5%). In Germany, compassion towards others was the strongest positive predictor (2%), while psychological distress had a strong negative impact (3%). Results highlight the importance of promoting emotional well-being to enhance vaccination coverage.

## Introduction

Four years into the COVID-19 pandemic, mass vaccinations have largely controlled the burden of the pandemic but have not prevented the spread of COVID-19 and other virus infections (Vasileiou et al., 2021). As the COVID-19 pandemic appears to be moving towards an endemic phase, concerns persist about the emergence of new virus variants that could disrupt the possibility of a complete return to fully functional and normal ways of life (Fernandes et al., 2022). These challenges warrant the need for continuous exploration of avenues, including attitudes towards COVID-19 vaccination, to eradicate the pandemic and any other emerging variants. Vaccination is recognised as one of the most cost-effective interventions in public health (Black and Richmond, 2018). Internationally, vaccination has been pivotal in the fight against numerous infectious diseases, including the devastating COVID-19 pandemic (Agrawal et al., 2021). It is estimated that 2–3 million deaths are prevented every year through vaccination in general, and it is expected that 122 million deaths will be averted through various vaccination programmes over the lifetime of individuals born between 2000 and 2030, according to the Global Alliance for Vaccines and Immunisation (GAVI; Gavi, 2020). The ongoing COVID-19 vaccination programme has resulted in substantial reduction in the negative impact of COVID-19 (Agrawal et al., 2021). Although, high COVID-19 vaccine hesitancy attitudes persist across diverse populations (Adu et al., 2023b).

Since the emergence of the COVID-19, several health behaviour models, such as the Health Belief Model (HBM), Theory of Planned Behaviour (TPB) and Ecological Model (EM), have predominantly been used to investigate attitudes and behaviours regarding COVID-19 vaccination and prevention measures (Adu et al., 2023b). These models have highlighted various components, including self-efficacy, perceived behavioural control and perceived susceptibility to COVID-19, as factors influencing COVID-19 prevention behaviours, including willingness to vaccinate against COVID-19 (Adu et al., 2023b; Breakwell et al., 2022). The impact of psychological and emotional states on health outcomes have also been documented in the literature. For instance, research has shown that positive affect is associated with better health behaviours, such as adherence to medication (Sin et al., 2015). Dispositional optimism, which involves a generalised and relatively stable positive outlook of life, has been linked to healthy lifestyle practices such as regular physical exercise (Lipowski, 2012). Additionally, self-compassion, defined as a non-judgemental attitude towards oneself, was associated with self-care behaviours, including healthy eating habits and better quality of sleep (Neff, 2003). Furthermore, studies have indicated that intentions to vaccinate against influenza were higher among individuals with self-perceived higher social status (Nowalk et al., 2015), and compassion towards others associated with reduced tendency to engage in excessive alcohol use amongst a Finish sample (Gluschkoff et al., 2019).

On the other hand, research indicated that stress symptoms negatively associated with COVID-19 vaccine uptake in both Germany and the USA (Brailovskaia et al., 2021). Similarly, religiosity has been identified as a factor influencing vaccine hesitancy, with beliefs in the existence of a supernatural or divine being impacting attitudes towards achieving herd immunity (Garcia and Yap, 2021; Huber and Huber, 2012). These findings highlight the importance of exploring the impact of emotional and psychological factors on health outcomes, including vaccination attitudes. Additionally, the above findings provide support for the Affect Infusion Model (AIM).

The AIM offers a robust conceptual foundation for explaining the psychological and emotional influences on individuals’ attitudes towards health behaviours (Forgas, 2013). The AIM posits that individuals’ mental processes and decision-making can significantly be influenced by their emotional states. The AIM further suggests that emotional responses may play an essential role in shaping individuals’ attitudes and intentions (Forgas, 1994). Thus, according to this model, an individual experiencing heightened favourable emotions tends to recall and prioritise positive concepts over negative ones, leading to a tendency to show more positive attitudes about current events compared to an individual with poor emotional well-being. This model prompts an exploration into how affective experiences influence health related attitudes such as vaccination (Forgas, 2013).

Notwithstanding, the above evidence revealed that the influence of emotional and psychological states on COVID-19 vaccination attitudes remains unclear in the literature. In other words, the affect infusion framework has not been used in previous COVID-19 vaccination attitudes studies. Further, the majority of the evidence was found in countries classified as Western, Educated, Industrialised, Rich and Democratic (WEIRD; Henrich et al., 2010). There were are also few cross-country studies regarding COVID-19 vaccination attitudes. Furthermore, a major methodological pitfall found in the extant literature was that the majority of the studies failed to provide a clear understanding of the magnitude of importance attributed to each of these variables. For instance, Brailovskaia et al. (2021) reported that all predictor variables including depression, stress, positive mental health, age and gender accounted for 12.9% of the explained variance in COVID-19 vaccine uptake among a sample of Chinese people, while Gluschkoff et al. (2019) reported only associations between compassion towards others and health behaviours.

In fact, variables have different explanatory capacities, suggesting that public health related measures towards promoting high COVID-19 vaccination coverage can be optimised when the unique variance in COVID-19 vaccination attitudes explained by each predictor variable is known. Therefore, by adopting the AIM, we assessed the unique contributions of psychological and emotional state variables, encompassing compassion towards others, dispositional optimism, religiosity, psychological distress, positive affect, self-perceived social status, and self-compassion in explaining the variability in COVID-19 vaccination attitudes among individuals living in Ghana, Germany, New Zealand, and India.

## Method

### Participants

The current study recruited 1822 participants from the general populations of Ghana (*n* = 523), Germany (*n* = 475), India (*n* = 411) and New Zealand (*n* = 413). Participants from India and Ghana were not rewarded for their participation in this study, as researchers from these countries relied of their large social networks for the data collection. Participants from New Zealand and Germany were given incentives by the Qualtrics data collection company for their participation. The ages of the participants ranged from 18 to 80 years in India (*M*_age_ = 26.14; SD = 8.57), 18 to 89 years in New Zealand (*M*_age_ = 46.35; SD = 18.07), 18 to 63 years in Ghana (*M*_age_ = 29.48; SD = 5.69) and 18 to 87 years in Germany (*M*_age_ = 44.09; SD = 5.57). In New Zealand, 275 participants (67%) identified as Europeans, 36 (9.2%) as Asians, 64 (16%) as Māori and Pacific, and 4 (1%) as Middle Eastern, Latin American and African (MELAA). Among the German sample, the majority of the participants identified as Germans (*n* = 396; 83%), while a smaller portion represented other nationalities (*n* = 79; 17%). All participants from India and Ghana identified themselves as belonging to their corresponding heritage. In Ghana, 338 participants (65%) were males, while 185 (35%) were females. In Germany, 223 (48%) identified as males, and 244 (52%) identified as females. Among the Indian participants, 130 (30%) were males, and 281 (68%) were females. In New Zealand, 179 (43%) were males, and 234 (57%) were females.

An a priori power analysis using G*Power 3.1 software was conducted to determine the required sample size for linear regression analysis with seven predictors. With an effect size of 0.15, an *α* error probability of 0.05 and an anticipated power of 0.95, the analysis yielded a required sample size of *n* = 153 for these parameters (Faul et al., 2009). Hence, the current sample was larger which contributed to increased statistical power.

### Procedure

Ethics approval for the current study was obtained from the authors’ institutional ethics committee. Convenience sampling via snowballing was used to collect data from Ghana and India using *SelectSurvey.net* data collection software. Data from these two countries were collected through various social media platforms such as WhatsApp, Email, Intagram, and Facebook. While online data collection methods have some limitations, they are found to be cost-effective and provide access to a broader segment of the population across diverse geographic locations (Lefever et al., 2007). For instance, we were able to obtain samples from all the 16 administrative regions in Ghana. We engaged the services of Qualtrics, a data collection company, to collect the data in Germany and New Zealand. Participation in this study was voluntary, and individuals aged 18 and above were eligible to participate in the study. The questionnaire included demographic factors such as age, biological sex, and ethnicity, and the main psychological measures. On average, participants took 15 minutes to complete the questionnaire.

### Measures

The 9-item version of the Motors of COVID-19 Vaccination Acceptance Scale (MoVac-COVID19S; Chen et al., 2022) was used to measure COVID-19 vaccination attitudes. This scale is available in both English and German (Adu et al., 2023c). This self-report instrument utilises a 7-point scale, ranging from 1 = ‘*Strongly agree*’ to 7 = ‘*Strongly disagree*’. An example item on this scale includes: ‘vaccination is a very effective way to protect me against COVID-19’. The scale demonstrated excellent reliability in the current study for India (Cronbach’s alpha (*α*) = 0.91; McDonald’s omega (*ω*) = 0.93, *M* = 44.14, SD = 10.61), New Zealand (*α* = 0.95, *ω* = 0.95; *M* = 46.35, SD = 13.20), Ghana (*α* = 0.94, *ω* = 0.94; *M* = 40.02, SD = 13.16), and Germany (*α* = 0.95; *ω* = 0.95; *M* = 43.26, SD = 0.677).

The Depression Anxiety Stress Scale (DASS-21; Lovibond and Lovibond, 1995) was used to measure psychological distress. The scale comprises 21 items and uses a 4-point Likert scale, ranging from 0 = ‘*Did not apply to me at all*’ to 3 = ‘*Applied to me very much*’. Sample items on the scale include: depression (‘I couldn’t seem to experience any positive feeling at all’), anxiety (‘I was aware of dryness in my mouth’), and stress (‘I found it hard to wind down’). The internal consistency coefficient of the scale in the current study was excellent for all countries: India (*α* = 0.94, *ω* = 0.94; *M* = 43.70, SD = 4.26), New Zealand (*α* = 0.97, *ω* = 0.97; *M* = 25.19, SD = 19.70), Ghana (*α* = 0.95, *ω* = 0.95; *M* = 35.01, SD = 12.83), and Germany (*α* = 0.96, *ω* = 0.96; *M* = 19.79, SD = 15.23). To mitigate multicollinearity, this scale was treated as a unidimensional measure (Medvedev et al., 2018). This involved calculating a single score by summing the responses to all items.

Positive affect was measured using the 10-item subscale of the Positive and Negative Affect Schedule (PANAS; Watson et al., 1988). Each adjective on this intrument is rated on a scale from 1 = ‘*Very slightly*’ to 5 = ‘*Extremely*’. Examples of adjectives measuring positive emotions include ‘interested’, ‘strong’ and ‘proud’. The reliability coefficient for this subscale in the current study ranged from very good to excellent: India (*α* = 0.88, *ω* = 0.88; *M* = 32.94, SD = 7.64), Ghana (*α* = 0.88, *ω* = 0.88; *M* = 37.66, SD = 7.32), New Zealand (*α* = 0.90, *ω* = 0.90; *M* = 30.86, SD = 7.82) and Germany (*α* = 0.90, *ω* = 0.90; *M* = 30.28, SD = 7.84).

We used the 12-item Self-Compassion Scale-Short Form (SCS-CF; Raes et al., 2011) to measure self-compassion. This self-reported scale is rated on a 5-point Likert scale from 1 = ‘*Never*’ to 5 = ‘*Always*’. A sample item on the scale include: ‘I try to see my failings as part of the human condition’. The scale demonstrated acceptable internal consistency in the present study for India (*α* = 0.70, *ω* = 0.71; *M* = 37.59, SD = 6.54), New Zealand (*α* = 0.74, *ω* = 0.71; *M* = 37.07, SD = 6.38), Germany (*α* = 0.71, *ω* = 0.75; *M* = 38.08, SD = 6.80), and very good reliability in Ghana (*α* = 0.81, *ω* = 0.81; *M* = 38.29, SD = 3.94).

Religious importance was assessed using the Centrality of Religiosity Scale (CRS-10; Huber and Huber, 2012). This scale has been used internationally (Adu et al., 2021, 2023a). An example of this 10-item self-reported scale include: ‘To what extent do you believe that God or something divine exists?’ Participants responses on the scale are rated on a 5-point Likert scale, ranging from 1 = ‘*Not at all*’ to 5 = ‘*Very much so*’. The internal consistency of the scale was excellent in India (*α* = 0.92; *ω* = 0.92, *M* = 9.30, SD = 35.14), New Zealand (*α* = 0.91; *ω* = 0.91, *M* = 23.83, SD = 10.97), Ghana (*α* = 0.90; *ω* = 0.90, *M* = 41.91, SD = 7.37), and Germany (*α* = 0.94; *ω* = 0.95, *M* = 20.70, SD = 9.98).

Compassion towards others was measured using the Santa Clara Brief Compassion Scale (SCBCS; Hwang et al., 2008). This 5-item measure is scored on a 7-point Likert scale, ranging from 1 = ‘*Not at all true of me*’ to 7 = ‘*Very true of me*’. Sample item found in the scale is: ‘I tend to feel compassion for people, even though I do not know them’. The scale exhibited excellent reliability in Ghana (*α* = 0.91; *ω* = 0.91, *M* = 27.09, SD = 6.94), and New Zealand (*α* = 0.91; *ω* = 0.91, *M* = 6.07, SD = 25.19), very good reliability in India (*α* = 0.84; *ω* = 0.82, *M* = 26.99, SD = 35.14), and low reliability in Germany (*α* = 0.45; *ω* = 0.48, *M* = 6.07, SD = 25.19). The limited number of items in the adapted German version of this scale may have resulted in the low reliability (Lee et al., 2016), as only three items showed acceptable loading on the latent variable during our confirmatory factor analysis. The other two items which showed negative factor loadings were removed to improve the statistical fit of the model.

Dispositional optimism was measured using the revised version of the Life Orientation Test (LOT-R; Scheier et al., 1994). This scale consists of 10 items, and it is rated on a 5-point Likert scale from 0 = ‘*Strongly disagree*’ to 5 = ‘*Strongly agree*’. Sample item on the scale is ‘In uncertain times, I usually expect the best’. The scale showed very good internal consistency in Ghana (*α* = 81; *ω* = 81, *M* = 16.47, SD = 2.66), and New Zealand (*α* = 0.83; *ω* = 0.83, *M* = 4.39, SD = 15.98), and acceptable reliability in Germany (*α* = 0.74; *ω* = 0.74, *M* = 15.79, SD = 3.96), and India (*α* = 0.60; *ω* = 0.62, *M* = 16.01, SD = 3.31).

Self-perceived social status was measured using the MacArthur Scale of Subjective Social Status (MacArthur SSS Scale; Adler et al., 2000). This single-item measure presents participants with a ladder containing 10 rungs, and they are asked to indicate their perceived position in society. A mark on the top rung represents a higher self-perceived social status, while a mark on the bottom rung represents a lower social status. The means and SDs of the scale are follows: Ghana (*M* = 2.03, SD = 6.27), New Zealand (*M* = 5.50, SD = 1.97), Germany (*M* = 5.41, SD = 1.92), and India (*M* = 6.50, SD = 2.01).

### Statistical analyses

#### Data preparation

We used the Statistical Package for the Social Sciences (IBM SPSS, version 28) to check for patterns of data missingness. The Missing Completely at Random (MCAR) test (Little, 1988) indicated that data from India and Ghana were not missing at random (MCAR, *p* < 0.05). Therefore, the Expectation Maximisation (EM) algorithm data imputation technique was employed to handle the few missing data points. In the case of Germany, the data was found to be missing completely at random (*p* = 0.22), justifying the use of the median of the available cases for each variable to replace the missing values (Huisman, 2000). There was no missing data in the New Zealand sample. All other analyses were conducted using Jeffreys’s Amazing Statistics Program (JASP Team, 2022). Descriptive analyses were conducted for demographic variables. Regarding the main analysis, the total scores of all non-binary scales were computed. Q-Q plots, skewness and kurtosis (within the acceptable range of −2 to +2; George, 2011) of the non-binary variables indicated that the variables were normally distributed. The assumption of homoscedasticity was met, as the casewise diagnoses revealed that all predicted values fell within a narrow range, indicating a non-significant difference in the spread of residuals across the range of predicted values. The scatterplot also showed randomly scattered points of variables around the horizontal axis. The Variance Inflation Factor (VIF) of all variables was below 5, suggesting the absence of multicollinearity (Marcoulides and Raykov, 2019).

#### Main analysis

Pearson’s correlation coefficients were computed for all the variables. This was followed by hierarchical multiple regression while controlling for country of residence. Afterwards, we conducted stepwise linear regression, which utilises both forward and backward entry methods. The modelling process starts with the inclusion of the strongest predictor that exhibits a substantial explained variance. The variance attributed to this predictor is then controlled, and subsequently, additional significant predictors that independently contribute to the outcome’s variance are sequentially incorporated. Predictors are entered (*p* < 0.05) or removed (*p* ≥ 0.05) one after the other. This procedure ensures the elimination of redundant variables to prevent the suppression of valid predictors due to shared variance (Medvedev et al., 2018; Roemer et al., 2021). We presented both overarching patterns and country-specific results. Our approach provides a nuanced understanding of variations across diverse geographical regions, enabling tailored strategies and interventions that address specific contexts and challenges in each country (Andrade, 2019; Medvedev et al., 2018).

## Results

Table 1 shows the results of Pearson’s correlations between study variables. The strongest correlations between COVID-19 vaccination attitudes and self-compassion (*r* = 0.81), followed by COVID-19 vaccination attitudes and dispositional optimism (*r* = 0.72) were found among the Indian participants. Similar positive associations were observed in Ghana, Germany and New Zealand, but with a lower magnitude (*r* = 0.16–0.26). On the other hand, psychological distress was negatively associated with COVID-19 vaccination attitudes in all the countries (*r* range: −0.09 to −0.39). Religiosity demonstrated a positive yet weak correlation with COVID-19 vaccination attitudes in Ghana (*r* = 0.22) and India (*r* = 0.18). However, it exhibited a weak negative correlation with COVID-19 vaccination attitudes in Germany (*r* = −0.18) and New Zealand (*r* = −0.08). Lastly, self-perceived social status exhibited a positive but weak relation with COVID-19 vaccination attitudes in India only (*r* = 0.24).

**Table 1.** Pearson’s correlation matrix between the psychological factors and COVID-19 vaccination attitudes in Ghana (*n* = 523), Germany (*n* = 475), New Zealand (*n* = 413), and India (*n* = 411).

Variables	Ghana	Germany	New Zealand	India
	COVID-19 vaccination attitudes	COVID-19 vaccination attitudes	COVID-19 vaccination attitudes	COVID-19 vaccination attitudes
Dispositional optimism	0.26**	0.16**	0.21**	0.72**
Compassion towards others	0.23**	0.21**	0.09*	0.16**
Religiosity	0.22**	−0.18**	−0.08	0.18**
Self-compassion	0.26**	0.19**	0.16**	0.81**
Psychological distress	−0.09*	−0.23**	−0.21**	−0.39**
Positive affect	0.27**	0.11*	0.15**	0.48**
Self-perceived social status	0.07	0.05	0.01	0.24**

* *p* < 0.05. **p < 0.01.

The results of the hierarchical multiple regression analysis (Table 2) revealed that, at the first step, country of residence significantly contributed to the regression model, *F*(1, 818) = 66.93, *p* < 0.001, explaining 10% of the variance in COVID-19 vaccination attitudes. Subsequently, introducing all the psychological variables in the second step accounted for an additional 9% of the variance in COVID-19 vaccination attitudes beyond the country effects. This change in *R*^2^ was statistically significant, *F*(1, 811) = 28.42, *p* < 0.001. Further, our results showed significant differences in COVID-19 vaccination attitudes among residents of in the countries. Compared to residents of India (baseline category), individuals in New Zealand, Germany, and Ghana reported significantly more favourable attitudes towards COVID-19 vaccination (Table 2). After controlling for country of residence, the results showed that positive affect, self-compassion, compassion towards others, and dispositional optimism directly related to COVID-19 vaccination attitudes. However, religiosity, self-perceived social status, and psychological distress negatively predicted COVID-19 vaccination attitudes (Table 2).

**Table 2.** Hierarchical multiple regression analysis of the psychological factors associated with COVID-19 vaccination attitudes in Ghana (*n* = 523), Germany (*n* = 475), New Zealand (*n* = 413), and India (*n* = 411).

Model	Variables	*β*	SE	*t*	*p*	*R* ^2^	*Δ* *R* ^2^
Model 1	Constant	–	0.61	57.34	<0.001	*0.10*	*0.10*
	New Zealand	0.37	0.86	13.45	<0.001		
	Germany	0.29	0.83	10.18	<0.001		
	Ghana	0.18	0.81	6.42	<0.001		
	India (baseline)	–	–	–	–		
Model 2	Constant	–	4.10	2.20	0.03	*0.18*	*0.09*
	New Zealand	0.58	2.50	7.19	<0.001		
	German	0.56	2.45	6.75	<0.001		
	Ghana	0.43	2.44	5.09	<0.001		
	Self-perceived social status	−0.02	0.15	−0.79	0.43		
	Positive affect	0.07	0.04	2.46	0.01		
	Self-compassion	0.27	0.05	3.52	<0.001		
	Compassion towards others	0.16	0.05	5.43	<0.001		
	Religiosity	−0.10	0.03	−3.19	<0.01		
	Dispositional optimism	0.12	0.10	4.44	<0.001		
	Psychological distress	−0.08	0.02	−2.64	0.01		

Table 3 presents the results of the stepwise linear regression predicting COVID-19 vaccination attitudes across the four countries. In India, 83% of COVID-19 vaccination attitudes was explained by the study predictors with self-compassion identified as the strongest positive predictor, explaining 66% of the variability. Dispositional optimism was the second strongest predictor which accounted for additional 16% of the variance. Self-perceived social status accounted for an additional 1% of variance, while religiosity and positive affect displayed a negligible but significant contribution (Table 2). In Ghana, 14% of COVID-19 vaccination attitudes was explained by the predictors with positive affect emerged as the strongest direct predictor, explaining 7% of the variability. This was followed by dispositional optimism, which accounted for 4% of the variability in COVID-19 vaccination attitudes.

**Table 3.** Summary of the stepwise multiple linear regression analysis of the psychological factors associated with COVID-19 vaccination attitudes in Ghana (n = 523), Germany (n = 475), New Zealand (n = 413), and India (n = 411).

Country	Outcome variable	Blocks	Predictors	*R* ^2^	Δ*R*^2^	β	*p*
Ghana	COVID-19 vaccination attitudes	1	Positive affect	0.07	0.07	0.14	0.003
		2	Dispositional optimism	0.10	0.04	0.13	0.006
		3	Self-compassion	0.12	0.02	0.15	<0.001
		4	Religiosity	0.13	0.01	0.10	0.031
Germany	COVID-19 vaccination attitudes	1	Psychological distress	0.03	0.03	−0.20	<0.001
		2	Compassion towards others	0.05	0.02	0.23	<0.001
		3	Religiosity	0.06	0.01	−0.13	0.005
New Zealand	COVID-19 vaccination attitudes	1	Dispositional optimism	0.04	0.05	0.15	0.009
		2	Psychological distress	0.05	0.01	−0.12	0.052
		3	Compassion towards others	0.06	0.01	0.13	0.012
		4	Religiosity	0.07	0.01	−0.12	0.038
India	COVID-19 vaccination attitudes	1	Self-compassion	0.65	0.66	0.60	<0.001
		2	Dispositional optimism	0.82	0.16	0.44	<0.001
		3	Self-perceived social status	0.82	0.01	0.06	0.003
		4	Religiosity	0.82	0.00	−0.07	0.002
		5	Positive affect	0.83	0.00	0.06	0.015

Self-compassion and religiosity, also contributed to the variance in COVID-19 vaccination attitudes, accounting for merely 2% and 1%, respectively. Among the New Zealand sample, 8% of the variance in COVID-19 vaccination attitudes was accounted for by the predictors. Among these predictors, dispositional optimism emerged as the strongest direct factor, explaining 5% of the variance. Compassion towards others, and religiosity explained an additional 1% each of the variance. Psychological distress inversely explained 1% of the variability in COVID-19 vaccination attitudes. In Germany, the study predictors explained only 6% of the variance in COVID-19 vaccination attitudes with psychological distress identified as the strongest inverse predictor of COVID-19 vaccination attitudes, explaining 3% of the total variance, followed by religiosity, accounting for 1% of the variance; and compassion towards others, directly accounted for 2% of the variability (Table 2).

## Discussion

We investigated the relation between psychological and emotional factors, and COVID-19 vaccination attitudes across four countries, whilst accounting for distinct contributions of the predictors to COVID-19 vaccination attitudes. Findings suggested that individual psychological factors, after controlling for country of residence, played a significant role in shaping COVID-19 vaccination attitudes. Individuals who showed higher levels of positive affect, self-compassion, compassion towards others, and dispositional optimism were more likely to have favourable attitudes towards COVID-19 vaccination. However, religiosity, self-perceived social status and psychological distress related negatively to attitudes towards COVID-19 vaccination. Further, our findings revealed distinct patterns in terms of specific variances accounted by the predictor variables across the different countries. Notably, in India, a substantial 83% of these attitudes were accounted for by the study predictors, with self-compassion emerging as the strongest and positive predictor. Conversely, in Ghana, 14% of the variance in COVID-19 vaccination attitudes was explained by the predictors, where positive affect was noted as the strongest direct predictor. In New Zealand, the predictors explained 8% of the variance, with dispositional optimism being the strongest direct predictor. Comparatively, in Germany, the study predictors explained merely 6% of the variability in COVID-19 vaccination attitudes and psychological distress notably emerged as the strongest inverse predictor. Religiosity accounted for a small proportion of the explained variance in COVID-19 vaccination attitudes, directly in Ghana, and indirectly across Germany, New Zealand and India.

The willingness to accept COVID-19 vaccination varied among the studied countries. New Zealand and Germany had higher acceptance rates of 75% and 74%, respectively, which align with previous studies (Neumann-Böhme et al., 2020; Thaker and Floyd, 2021). In contrast, Ghana and India had lower acceptance rates of 58% and 55%, respectively. These findings are consistent with previous findings which indicated lower vaccination rates in developing countries compared to WEIRD countries (Nehal et al., 2021). Overall, our findings regarding the psychological factors and COVID-19 vaccination attitudes suggest that emotional well-being is important for fostering positive attitudes towards COVID-19 vaccination and potentially other infectious diseases. This is in line with the AIM’s assumptions (Forgas, 1994). The positive impact of self-compassion on COVID-19 vaccination attitudes highlights the relevance of self-care in shaping these attitudes, an observation supported by studies demonstrating that self-compassionate individuals tend to engage in protective behaviours during pandemics (Mohammadpour et al., 2020).

The direct link between positive affect and COVID-19 vaccination attitudes aligns with prior research that found positive association between positive emotional states and health-promoting behaviours (Shiota et al., 2021; Sin et al., 2015). This positive affect-COVID-19 vaccination attitudes relation means that individuals with higher levels of positive emotions are more likely to have favourable attitudes towards COVID-19 vaccination. This finding underscores the role of emotions in shaping vaccinations attitudes. The positive link between dispositional optimism and COVID-19 vaccination attitudes agrees with previous research which found that positive expectations towards life influence health-related attitudes (Carver et al., 2010). Similar reports indicate that dispositional optimism was associated with regular exercises and lower levels of distress (Lipowski, 2012), supporting our findings. Additionally, the significance of compassion towards others in contributing to better vaccination attitudes emphasises the role of caring for others in health behaviours. Research has shown that compassion towards others has psychological and physical health benefits (Gluschkoff et al., 2019).

Nevertheless, the inverse relation between psychological distress and COVID-19 vaccination attitudes indicates that individuals experiencing higher levels of psychological distress are less likely to have favourable attitudes towards COVID-19 vaccination. This finding highlights the impact of mental health on health-related attitudes. Research has consistently indicated the detrimental effect of distress on diverse areas of life (Brailovskaia et al., 2021). The negative relation between self-perceived social status and COVID-19 vaccination attitudes demonstrates that as self-perceived social status increases, vaccination attitudes become less favourable, contrasting previous research on vaccination attitudes (Bodelet et al., 2021). While Bodelet et al. (2021) found a positive association between self-perceived social status and flu vaccination attitudes in France, we observed a negative association with COVID-19 vaccination attitudes. This discrepancy in the findings may be attributed to the differences in the contexts of the two studies and the circumstances surrounding these diseases, highlighting the importance of contextual research on specific pandemic and epidemics. We also found that COVID-19 vaccination attitudes decreased with increased religiosity. This is consistent with existing literature documenting the negative impact of religiosity on vaccination in general (Wombwell et al., 2015). A study examining data from 144 regions worldwide also showed that religiosity was inversely related to COVID-19 vaccination rates (Martens and Rutjens, 2022). Religious beliefs such as fatalism, faith healing, and divine protection have been identified as factors that impede efforts to promote high vaccination rates (Mamani-Benito et al., 2023).

Specifically, the variances accounted by the predictor variables across the countries imply a noteworthy trend. For instance, while the psychological factors exerted substantial impact on COVID-19 vaccination attitudes in India, the scenario in Germany showcased nearly negligible predictive power. This discrepancy becomes particularly intriguing when considering the high COVID-19 vaccination willingness rate in Germany. Such a pattern raises the possibility that distinct motivational mechanisms are at play in each country. In Germany, where compliance with government policies appears stronger, conformity to these policies might predominantly drive vaccination intentions. Conversely, in India, the dominance of psychological factors suggests the possible critical role of these factors in shaping vaccination attitudes, making public health measures towards enhancing positive emotional states vital for promoting high vaccination coverage. Such finding can inform policies in countries with relaxed immunisation policies or limited government emergency powers to promote compliance.

Recognising these country-specific dynamics underscores the need for tailored measures to optimise COVID-19 and other vaccination acceptance rates, accounting for the varying motivational drivers and cultural contexts present in different regions. For instance, in the current study, country-specific results showed a positive relation between religiosity and COVID-19 vaccination attitudes in Ghana, but the reverse was the case in other countries. This could imply that in a highly religion country like Ghana, where places of worship (e.g. churches) are used to educate and promote health especially during disease outbreaks, this could potentially result in direct relation between religiosity and vaccination attitudes. However, in contexts where religious institutions are not positively involved in health promotion, the association between religiosity and vaccination attitudes may be different, possibly resulting in a negative relation between COVID-19 vaccination attitudes for Germany, India, and New Zealand respondents (Anshel and Smith, 2014).

### Strengths and limitation

We are the first to investigate this combination of psychological variables in relation to COVID-19 vaccination attitudes across four countries within a single study. The use of stepwise regression analysis has provided a comprehensive understanding of the relative importance of each variable in contributing to COVID-19 vaccination attitudes across the countries. Our study contributes to the limited literature on vaccination attitudes in Low-Middle, and high-income countries, thus mitigating the over reliance on evidence from unrelated contexts (Henrich et al., 2010). However, there are certain limitations to this study. Firstly, the use of convenient sampling may introduce selection bias as researchers rely on a subset of the population that is readily available. Additionally, the use of online data collection may limit the findings of the study to the population that has internet access. Moreover, the cross-sectional design employed in this study does not allow for causal inferences. Self-report measures used in the study carry the risk of introducing bias, such as social desirability responses, and participants may make guesses about possible relations when responding to such measures (Grimm, 2010). Another inconsistency involved the slightly different approaches used in recruiting the samples (e.g. media platforms vs data collection company).

### Implications, and future directions

We have provided valuable insights for targeted measures to enhance COVID-19 vaccination attitudes by identifying the most impactful psychological factors across the countries. Overall, our results highlighted the advantages of nurturing positive psychological resources (e.g. Medvedev et al., 2018) to promote favourable attitudes towards COVID-19 vaccination and future pandemics. Particularly, addressing psychological distress among individuals hesitant about vaccination through the provision of mental health resources could help improve COVID-19 and possibly other vaccination rates. This approach may not only increase positive attitudes towards vaccination but also enhance antibody response to the vaccine (Madison et al., 2021). Policies promoting positive emotions and the need to care for others could create a supportive environment that encourages favourable vaccination attitudes. Notably, stakeholders should be aware of the cultural differences regarding emotional states. For instances, in collectivist cultures like India, interventions could focus on fostering positive social interactions and community cohesion to enhance positive affect. In contrast, in individualistic cultures like Germany, interventions may be more effective if they emphasise personal achievements and goal attainment (Mesquita, 2001). Understanding these nuances enhances the impact of interventions.

Healthcare providers could play a role by receiving training to address emotional and psychological factors in patient interactions regarding vaccination. This could involve empathetic communication and addressing concerns related to optimism and self-compassion. Efforts to improve COVID-19 and other vaccination rates should be sensitive to cultural and contextual elements, such as religiosity. Vaccination campaigns can be tailored to be compatible with religious teachings or working with religious leaders to promote vaccination within their communities (Adu et al., 2023b). Future research should consider employing a longitudinal and qualitative study design to establish causal relations between the various psychological variables and vaccination attitudes. This would allow for drawing tentative conclusions from the current results. Additionally, there is a need to investigate how cultural dynamics, such as collectivism, interact with emotional states to shape vaccination attitudes.

## Conclusions

Our study investigated the unique contribution of psychological factors, including compassion towards others, dispositional optimism, religiosity, psychological distress, positive affect, self-perceived social status, and self-compassion, to COVID-19 vaccination attitudes across four countries. Our findings suggest that individual psychological factors, in addition to country of residence, significantly influenced COVID-19 vaccination attitudes. Results further revealed that compassion towards others, dispositional optimism, positive affect, and self-compassion were the strongest direct predictors of COVID-19 vaccination attitudes in Germany, New Zealand, Ghana and India, respectively. On the other hand, psychological distress was found to be the strongest inverse predictor of COVID-19 vaccination attitudes in Germany. Religiosity had a relatively minor influence on COVID-19 vaccination attitudes across the countries. By understanding the specific contributions of these psychological factors, tailored measures can be established to promote vaccination efforts. Future research is required to explore the cause-and-effect links between these psychological factors, cultural dynamics, and health outcomes including vaccination attitudes.
